# An image‐based method to synchronize cone‐beam CT and optical surface tracking

**DOI:** 10.1120/jacmp.v16i2.5152

**Published:** 2015-03-08

**Authors:** Aurora Fassi, Joël Schaerer, Marco Riboldi, David Sarrut, Guido Baroni

**Affiliations:** ^1^ Dipartimento di Elettronica Informazione e Bioingegneria, Politecnico di Milano Milano Italy; ^2^ CREATIS, CNRS UMR 5220, INSERM U1044, Université Lyon 1, INSA‐Lyon Villeurbanne France; ^3^ Department of Radiotherapy Centre Léon Bérard Lyon France; ^4^ Bioengineering Unit, CNAO Foundation Pavia Italy

**Keywords:** synchronization, cone‐beam CT, optical surface, tumor tracking, external–internal correlation

## Abstract

The integration of in‐room X‐ray imaging and optical surface tracking has gained increasing importance in the field of image guided radiotherapy (IGRT). An essential step for this integration consists of temporally synchronizing the acquisition of X‐ray projections and surface data. We present an image‐based method for the synchronization of cone‐beam computed tomography (CBCT) and optical surface systems, which does not require the use of additional hardware. The method is based on optically tracking the motion of a component of the CBCT/gantry unit, which rotates during the acquisition of the CBCT scan. A calibration procedure was implemented to relate the position of the rotating component identified by the optical system with the time elapsed since the beginning of the CBCT scan, thus obtaining the temporal correspondence between the acquisition of X‐ray projections and surface data. The accuracy of the proposed synchronization method was evaluated on a motorized moving phantom, performing eight simultaneous acquisitions with an Elekta Synergy CBCT machine and the AlignRT optical device. The median time difference between the sinusoidal peaks of phantom motion signals extracted from the synchronized CBCT and AlignRT systems ranged between ‐3.1 and 12.9 msec, with a maximum interquartile range of 14.4 msec. The method was also applied to clinical data acquired from seven lung cancer patients, demonstrating the potential of the proposed approach in estimating the individual and daily variations in respiratory parameters and motion correlation of internal and external structures. The presented synchronization method can be particularly useful for tumor tracking applications in extracranial radiation treatments, especially in the field of patient‐specific breathing models, based on the correlation between internal tumor motion and external surface surrogates.

PACS number: 87

## I. INTRODUCTION

Accurate target localization is a crucial issue in modern conformal radiotherapy, aiming at the delivery of high radiation doses to the tumor volume while minimizing the irradiation of adjacent critical structures. Geometric errors due to breathing‐induced organ motion may significantly degrade the effectiveness of radiotherapy treatments for thoracic and abdominal tumors, such as in lung cancer.^(1)^ Image‐guided radiotherapy (IGRT) is widely applied in the treatment of extracranial tumors for the assessment of target position and for the compensation of inter‐ and intrafraction motion.^(2)^ Common IGRT modalities are represented by in‐room X‐ray imaging and optical surface tracking.^(3)^ In‐room imaging allows the direct localization of the tumor or implanted clips with high accuracy,^(4)^ but involves the invasive use of nontherapeutic ionizing radiation. An alternative for direct tumor tracking relies on implanted transponders detected by means of external electromagnetic receivers. However, the insertion of fiducials within the lung can be associated to the risk of pneumothorax or fiducial migration.^(5)^ Conversely, optical surface tracking provides noninvasive patient motion monitoring and can be applied for deriving a breathing surrogate from which tumor motion is indirectly estimated by means of external–internal correlation models.^(6)^ Optical localization devices are used to capture the displacement of the patient surface, by reconstructing the position of active or passive markers placed on the patient skin^(7)^ or by scanning the entire markerless surface.^8^, ^9^ A multidimensional respiratory motion signal can be extracted both from surface marker trajectories and from markerless optical surfaces by applying deformable mesh registration.^(10)^

The integration of in‐room X‐ray imaging and optical surface tracking has gained increasing importance in IGRT.^(11)^ A possible application is related to motion compensation by means of tumor tracking methods, encompassing the real‐time adaptation of the irradiation geometry as a function of the estimated changes in tumor position.^(12)^ Tumor tracking is clinically applied in photon radiotherapy by combining the optical localization of multiple surface markers with the X‐ray imaging of the tumor through orthogonal radiographies.^(13)^ A patient‐specific external–internal correlation model is initialized before treatment and periodically updated throughout the whole fraction by simultaneously acquiring the external surface surrogate and the internal tumor motion.^(14)^ The integrated approach overcomes the drawbacks of the single techniques: the noninvasivity of optical tracking allows the continuous monitoring of intrafraction organ motion during the entire treatment course, whereas anatomical data provided by X‐ray imaging enables the update of the external–internal correlation to reduce tumor position estimation uncertainties.^15^, ^16^ The accuracy of tumor tracking systems based on correlation models was quantified within 2.5 mm in the anteroposterior direction and 1.9 mm in the mediolateral and superior–inferior (SI) directions.^(17)^

A widespread in‐room imaging technique in high precision radiation treatment is cone‐beam computed tomography (CBCT), which provides a volumetric reconstruction of the patient internal anatomy from the acquisition of a sequence of two‐dimensional (2D) projections at different rotational angles.^(18)^ Three‐dimensional (3D) CBCT images are applied for the initial treatment setup to correct patient positioning errors and target misalignment by means of registration with the CT scan used for treatment planning. In order to reduce blurring artifacts caused by respiratory motion, a respiration‐correlated CBCT or a motion‐compensated CBCT can be reconstructed by using a breathing surrogate extracted directly from the acquired projections^19^, ^20^ or from external monitoring devices.^(21)^ Several methods have also been proposed to track tumor position in the rotational CBCT projections^22^, ^23^ and to reconstruct the 3D tumor trajectory during the whole scan,^(24)^ thus providing information on daily tumor motion patterns. Recently, a method was proposed to build a surrogate‐driven breathing motion model from a CBCT scan, which is envisaged for tumor tracking applications.^(25)^ The model describes tumor motion due to respiration over the entire breathing cycle and is driven by an external surface surrogate used for breathing phase identification. Breathing motion models can also be extracted from planning cine‐CT^(26)^ or four‐dimensional (4D) CT^(27)^ and then adapted to interfraction baseline variations based on daily CBCT imaging.^(28)^

An essential step for the integration of X‐ray imaging and optical tracking is the synchronization between the applied systems, in order to retrieve the temporal correspondence between the acquisition of X‐ray projections and optical surfaces.^(29)^ The synchronization can be achieved through external X‐ray detectors, such as the Blackcat (Blackcat, Westminster, MD), connected to the optical tracking device.^(25)^ The detectors sample at high frequency the photon count, whose peaks correspond to the acquisition of X‐ray images. Some surface tracking systems, such as the AlignRT (VisionRT, London, UK), can be directly synchronized by means of an hardware interface to the X‐ray machine.^(30)^ The acquired optical surfaces are fagged with the X‐ray on/off information provided by the interface. In this case, the temporal resolution of the synchronization is limited by the surface frame rate, which generally does not exceed 10 Hz for the scanning of the entire thoraco–abdominal surface.^26^, ^28^

The aim of this study was to develop an image‐based method to synchronize CBCT scan with optical surface acquisition, without requiring the use of additional devices or interfaces and overcoming the limitation associated with surface acquisition frequency. The proposed method relies on imaging data captured with the X‐ray and optical systems, combined with a precalibrated transfer function model. The developed synchronization approach was tested on a motorized moving phantom, performing simultaneous CBCT and optical acquisitions. The investigated method was also applied to clinical data acquired from seven lung cancer patients, in order to test the potential in estimating the individual breathing patterns and motion correlation of different internal and external structures.

## II. MATERIALS AND METHODS

### A. Image‐based synchronization method

We present an image‐based method for the temporal synchronization of CBCT and optical surface acquisitions, which can be generalized both to marker‐based and markerless surface tracking systems. The proposed synchronization approach does not require any additional hardware, but relies only on the imaging data captured by the CBCT and optical systems. In particular, the developed method consists of tracking with the optical device the motion of a component of the CBCT/gantry unit that rotates during the acquisition of the CBCT scan, such as the X‐ray source or the flat‐panel detector. The only condition for the applicability of the proposed synchronization approach is that the field of view (FOV) of the optical tracking system includes a moving CBCT/gantry component for a short time window during the acquisition of the CBCT scan. This condition is usually guaranteed with the standard mounting configuration of the optical tracking systems, which are commonly suspended from the treatment room ceiling in a frontal or lateral position with respect to the couch.^(8)^ The video images captured by the cameras of the optical system for marker or surface reconstruction can be used to track the position of the visible CBCT/gantry component. A calibration procedure is required to relate the position of the optically tracked rotating component with the gantry angle, which is univocally associated with the time elapsed since the beginning of the CBCT scan. This allows obtaining the temporal correspondence between the acquisition of X‐ray projections and optical surfaces. The application of the presented method to the specific CBCT and optical tracking systems available for this study is described in the following paragraphs.

**Figure 1:**
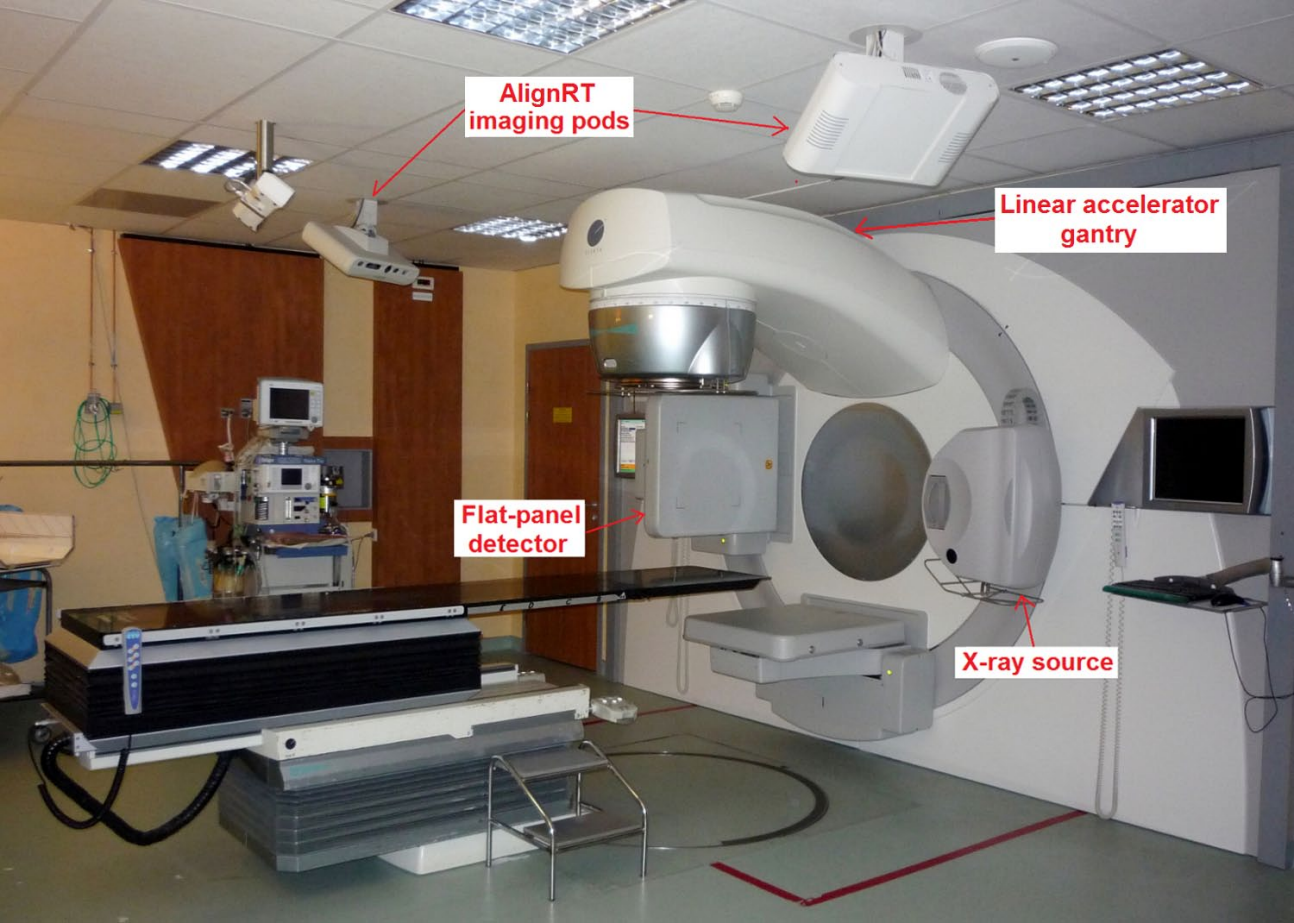
Treatment room at CLB where the Elekta Synergy and AlignRT systems are installed. The figure depicts the AlignRT camera pods for optical surface tracking and the X‐ray source and flat‐panel detector for CBCT imaging.

The proposed CBCT/optical synchronization method was implemented and tested at Centre Léon Bérard (CLB) in Lyon, France. The CBCT machine used in the present study is the Elekta Synergy (Elekta, Stockholm, Sweden), which is equipped with the XVI on‐board kV imaging system. As shown in Fig. 1, the CBCT X‐ray source and the flat‐panel detector are orthogonal to the treatment beam and rotate simultaneously with the linear accelerator gantry. The typical CBCT scan consists of approximately 650 projections acquired over a complete 360° gantry rotation with a frame rate of 5.5 Hz. The CBCT image sequence is acquired clockwise, starting with the X‐ray source at the left‐side of the treatment couch (gantry angle at ‐180°). As depicted in Fig. 1, at CLB the AlignRT (Vision RT, London, UK) surface tracking system is installed in the same treatment room of the Elekta Synergy CBCT machine. The AlignRT system is composed of two imaging pods suspended from the room ceiling in a symmetric lateral position, each one equipped with two charge‐coupled device (CCD) cameras for stereovision and a third camera for texture acquisition. A research version of AlignRT (GateCT) based on a single imaging pod was used in this study, since it allows fast acquisition of optical surfaces by means of the high speed image capture (HSIC) component. In particular, the pod positioned at the right‐side of the treatment couch was selected for the synchronization with the CBCT scan, providing a surface frame rate of about 8–9 Hz.

**Figure 2:**
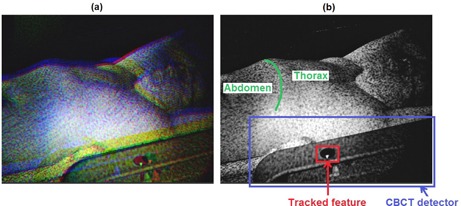
Video image (a) acquired by the right‐side imaging pod of the AlignRT system. The images captured by the three cameras of the pod are superimposed as RGB components. The slight offset between the three components is due to the different position of the cameras in the imaging pod. Blue image component (b), depicting the CBCT flat‐panel detector and the elliptic feature used to track the detector motion. The abdominal and thoracic regions of patient surface are also represented, highlighting the costal margin as separation line.

**Figure 3:**
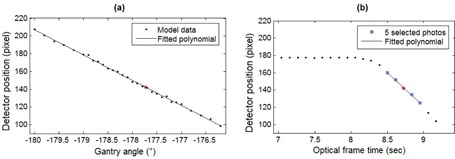
Calibrated transfer function model (a) relating the gantry angle with the detector position identified on the video images captured by the AlignRT system. Detector positions (b) obtained from AlignRT images at the beginning of a CBCT scan with a starting gantry angle of ‐180°. The position of the detector is not changing as a function of optical frame time in the first ten measurements since they were taken before the CBCT start. The five video images selected for CBCT/ optical synchronization are highlighted as blue squares. In panel (b), the red star corresponds to the detector position interpolated at the mean optical frame time of the five selected images, while the corresponding gantry angle is obtained from the fitted polynomial in panel (a).

An exemplificative video image acquired by the right‐side imaging pod of the AlignRT system at the beginning of a CBCT scan at ‐180° gantry angle is shown in Fig. 2(a). The three images captured simultaneously by the two stereo cameras and by the texture camera included in the pod are superimposed in a single image as different RGB (red, green, and blue) components. Since a portion of the CBCT flat‐panel detector was visible in the video images acquired by the selected pod (Fig. 2(b)), the tracking of the detector motion immediately after the CBCT start was exploited to synchronize the acquisition of CBCT and AlignRT systems. The tracking was performed on the blue image component extracted from the captured video images, which contains a larger visible portion of the panel. Figure 2(b) depicts the elliptic‐shaped feature inherent to the panel that was used to track the CBCT detector motion. Threshold‐based segmentation algorithms and ellipse fitting operations were applied to estimate the position of the selected feature on the AlignRT video images. A transfer function model was calibrated to relate the position of the CBCT detector identified in the AlignRT video images with the corresponding rotation angle of the gantry. As depicted in Fig. 3(a), model calibration was obtained by considering 30 rotational positions of the CBCT/gantry unit ranging from ‐180° to ‐176°, which correspond to the initial angles of the CBCT scan. For each rotational position, 50 video images were captured by the AlignRT system. The elliptic feature of the CBCT detector was identified on each image and the detector position was computed as the mean distance of the elliptic feature centre from the top‐left image corner. The transfer function model was obtained by fitting with a first order polynomial the estimated detector positions as a function of the gantry angle. As shown in Fig. 3(a), the linear fitting of the detector position is a good approximation for gantry rotations of limited entity.

According to the proposed synchronization method, five video images captured by the AlignRT system after the beginning of the CBCT scan were selected, as depicted in Fig. 3(b). The use of multiple video images allows increasing robustness against possible inaccuracies in segmenting the elliptic feature of the CBCT detector. These data were linearly interpolated to derive the detector position at the mean optical frame time (τ) of the five selected images.

The calibrated transfer function model was then applied to estimate the gantry angle associated with the interpolated detector position (Fig. 3(a)). The estimated gantry angle was related to the CBCT frame time (ε) by using the log file data stored by the Elekta Synergy system. The log file includes, for each acquired CBCT projection, the corresponding gantry angle and the time elapsed since the beginning of the CBCT scan. The temporal difference between CBCT and AlignRT acquisitions was computed as τ‐ε. By subtracting the estimated difference from the optical frame time, each captured surface was then tagged with the corresponding CBCT frame time, thus allowing the synchronization between the two imaging systems.

### B. Phantom evaluation

**Figure 4:**
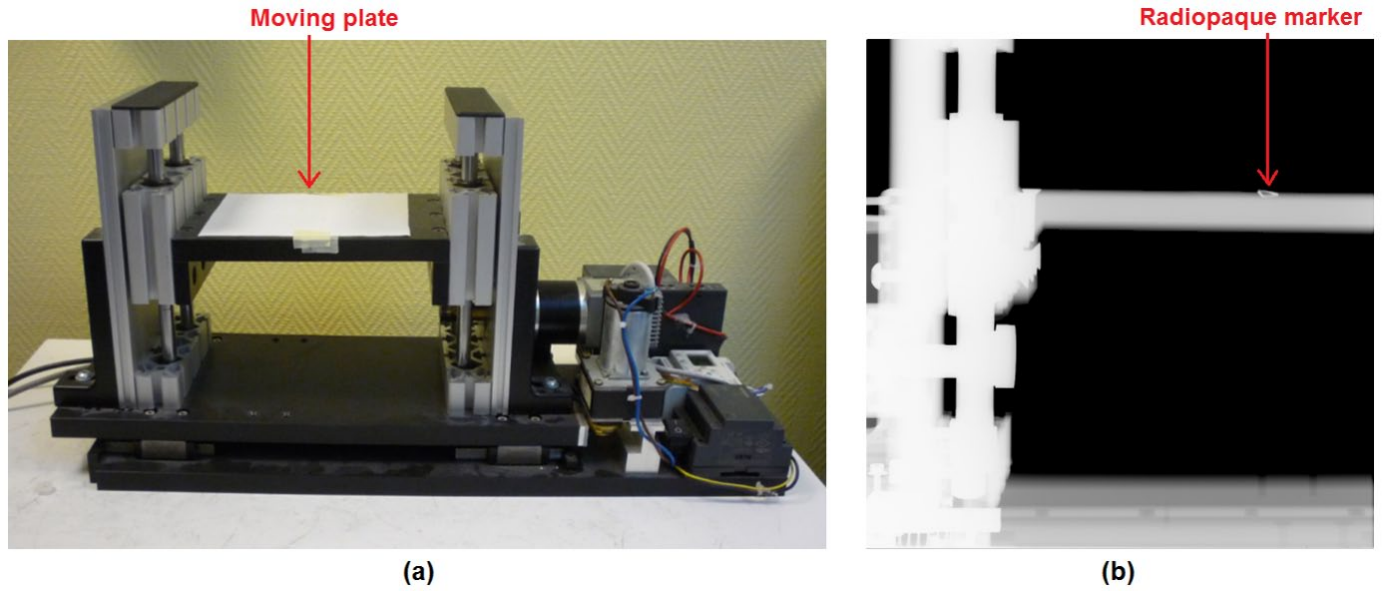
Motorized phantom (a) with moving plate used for testing the temporal accuracy of the proposed synchronization method. CBCT projection (b) of the phantom with the radiopaque marker used for motion tracking.

The time accuracy of the proposed image‐based method for the synchronization of CBCT and optical surface acquisitions was evaluated on a motorized moving phantom. As shown in Fig. 4(a), the phantom is composed of a horizontal plate moved vertically by a microprocessor‐controlled stepping motor along a sinusoidal trajectory, thus simulating the breathing motion of the thoraco–abdominal surface. Eight simultaneous CBCT and AlignRT acquisitions of the moving phantom were performed, setting the motion amplitude to 15 mm and varying the frequency from 0.75 to 1 Hz (Table 1). For each test, 50 CBCT projections were acquired, corresponding to about 9 s. The gantry angle associated to the acquired projections ranged between $-180^{\circ }$ and $-150^{\circ }$. In order to track phantom motion in CBCT projections, a radiopaque marker was placed on the horizontal plate (Fig. 4(b)) and threshold‐based segmentation algorithms were applied to identify the marker position in each CBCT image. The phantom motion signal was obtained as the trajectory of the segmented marker along the vertical dimension of CBCT projections. Phantom motion was simultaneously tracked with the AlignRT system by acquiring the 3D optical surfaces of the horizontal plate. The motion signal was extracted from the captured surfaces by computing the average vertical trajectory of the surface points included in a region of interest of the phantom plate encompassing the marker. The motion signals derived from CBCT projections and optical surfaces were analyzed after applying the proposed synchronization method. The signals were normalized and fitted to a sinusoidal curve using a non‐linear least‐squares approach. The timestamps associated with the maximum and minimum peaks of the two sinusoids were extracted and compared.

For each phantom test, the accuracy of the calibrated transfer function model relating the gantry angle with the CBCT detector position identified in the AlignRT video images (Fig. 3(a)) was also evaluated. In particular, the gantry angle at the beginning of the phantom CBCT scan was estimated with the calibrated model, by using the mean detector position extracted from the ten images captured before the starting of the CBCT acquisition. Multiple video images were used also in this case to reduce the influence of possible inaccuracies in identifying the CBCT detector. The estimated angle was compared with the real value of the initial gantry angle derived from the Synergy log file.

**Table 1:** Temporal and angular accuracy of the proposed synchronization method tested on the moving phantom. The temporal difference between the sinusoidal peaks of the motion signals extracted from CBCT projections and optical surfaces are expressed as median value ± interquartile range (IQR)

*Test*	*Frequency of Phantom Motion (Hz)*	*Temporal Accuracy (msec)*	*Angular Accuracy (°)*
T1	0.75	4.4±14.4	0.05
T2	0.85	12.9±2.9	0.07
T3	1.0	1.1±3.6	0.08
T4	1.0	1.2±0.2	0.07
T5	1.0	0.8±2.0	0.09
T6	1.0	2.8±5.1	0.09
T7	1.0	−3.1±5.5	0.04
T8	1.0	7.8±7.3	0.08
Median±IQR		2.0±5.2	0.08±0.03

### C. Patient testing

The proposed synchronization method was applied to a clinical database of seven early‐stage non‐small cell lung cancer patients with upper‐lobe tumors, treated at CLB with stereotactic body radiotherapy. All patients were male except patient P4, and the mean age was 76 yrs (range 65 to 85 yrs). Data collected for each patient included the displacement of the thoraco–abdominal surface captured with the AlignRT optical system during the first 120° of a CBCT scan. For the remaining angular positions, the rotating gantry and CBCT units occluded the patient surface to the right‐side imaging pod (Fig. 1). This occlusion problem, which did not interfere with the synchronization procedure, was due to the specific lateral positioning of the AlignRT system at CLB, but could be avoided with a frontal installation of the optical device. Since patient treatment sessions were performed from one up to four months after phantom tests and calibration of the transfer function model applied for synchronization (Fig. 3(a)), the acquired patient data were used to verify the long‐term validity of the calibrated model. Any change or update was done for the AlignRT system between phantom tests and clinical data tests. The synchronized patient database was also exploited to study the variations in the breathing motion patterns of different internal and external structures, which are commonly used as respiratory surrogates in lung radiotherapy.^(1)^ The considered surrogates included the diaphragm motion^(31)^ and the displacement of thoracic and abdominal surface regions (Fig. 2(b)).^(32)^ The correlation with respect to lung tumor motion was analyzed for each surrogate, taking into account the possible phase shift, which represents the time delay between the motion induced by respiration of external and internal structures.^31^, ^32^

The trajectory of lung tumors in the SI direction was obtained by segmenting the tumor in CBCT images with a semiautomatic approach, based on template matching algorithms applied to the contrast‐enhanced projections.^(33)^ Diaphragm motion projected along the SI axis was extracted from CBCT images by using the Amsterdam Shroud method^34^, ^35^ implemented in the open‐source reconstruction toolkit RTK.^(36)^ The external respiratory surrogates for thoracic and abdominal surface regions were extracted from the optical data acquired by the AlignRT system. A deformable surface registration algorithm was applied to derive the spatial correspondence between consecutive markerless optical surfaces, thus obtaining the 3D trajectories of surface points.^(10)^ Unlike rigid registration, the deformable approach allows capturing local surface transformations and complex breathing motion patterns, which generally vary for different regions of the thoraco‐abdominal surface. A monodimensional motion signal was derived for each surface point by computing frame‐by‐frame the Euclidean distance between the registered 3D coordinates and the most posterior position. K‐means clustering techniques were applied to all surface points' signals to separate the surface in two clusters, represented by the thorax and the abdomen (Fig. 2(b)). A single surrogate signal was obtained for each cluster by averaging the motion trajectories of all surface points belonging to the cluster.

For each patient, tumor and surrogate signals were filtered using a third‐order, low‐pass Butterworth filter, with a normalized cutoff frequency of 0.3. Baseline time trends were removed by subtracting a smoothed version of the signal, obtained with a moving average filter whose window size spread approximately over two respiratory cycles. For both tumor and surrogate signals, the motion amplitude was computed as the difference between the 95th and 5th percentiles of the baseline‐free signal. After data synchronization, the phase shift between tumor motion and each surrogate was obtained by translating the surrogate signal in the temporal dimension and finding the time delay that gave the highest correlation with the tumor signal.

## III. RESULTS

**Figure 5:**
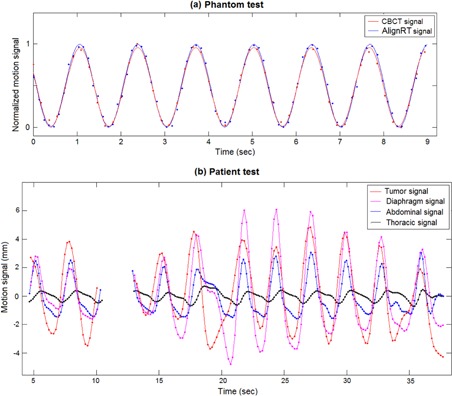
Sinusoidal fitting of phantom motion signals (a) estimated from CBCT projections and optical surfaces for test T1. Motion signals of different internal and external structures (b) extracted from synchronized CBCT and optical surface acquisitions for patient P1. The figure represents only CBCT images acquired without surface occlusion in which lung tumor could be identified.

For the first test performed on the moving phantom (T1), Fig. 5(a) shows the motion signals derived from the synchronized CBCT and optical surface acquisitions, fitted to sinusoidal curves. The temporal difference between the sinusoidal peaks of the two synchronized motion signals are reported in Table 1 for each phantom test. The median value of the measured differences ranged between ‐3.1 and 12.9 msec, with a peak interquartile range (IQR) of 14.4 msec. The differences between the real and estimated starting angles of the CBCT scan for each phantom test are also listed in Table 1. All angular differences were lower than 0.1°, with a median value of 0.08°. The long‐term accuracy of the calibrated model assessed on patient CBCT acquisitions is depicted in Table 2. The errors in estimating the CBCT starting angle varied from ‐0.1 to 0.2°, with a median absolute value of 0.06°. The real rotational positions of the treatment gantry at the beginning of patient CBCT scans are also listed in Table 2. The initial CBCT angles are lower than the nominal one $\left(-180^{\circ }\right)$, due to the mechanical limitations in gantry rotation.

**Figure 6:**
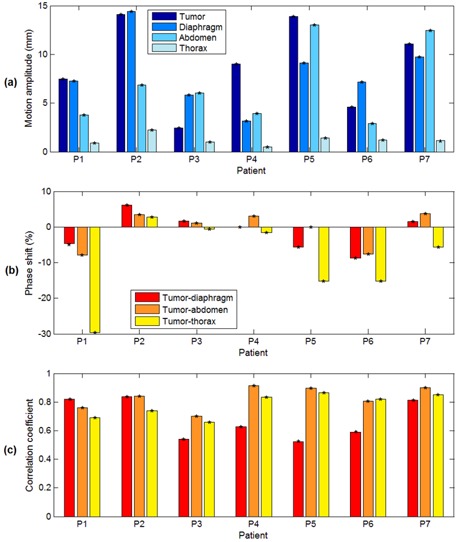
Motion amplitude (a) computed for the different internal and external structures. Percentage phase shifts (b) measured between tumor motion and breathing surrogates. Linear Pearson correlation coefficients (c) between tumor and surrogate signals, after compensating for the measured phase shifts.

About 20–35 s of synchronized CBCT/AlignRT acquisitions were analyzed for each patient (Table 2); only CBCT images acquired without surface occlusion were considered. As reported in Table 2, the number of breathing cycles per patient included in the synchronization period ranged between 5 and 9. The corresponding mean cycle lengths measured from tumor motion signals are also listed in the Table. The motion signals for the different internal and external structures extracted for patient P1 are shown in Fig. 5(b). Figure 6(a) depicts for each patient the motion amplitude computed for the considered structures. The phase shifts measured between tumor motion and surrogate signals, expressed as a percentage of the breathing cycle, is shown in Fig. 6(b). A large interpatient variability was obtained for motion amplitude and tumor‐surrogate phase shifts. Thoracic surface displacement showed the highest phase shifts with tumor motion, with values up to −29.6% for patient P1 (Fig. 5(b)). Conversely, absolute median phase shifts (±IQR) for diaphragm motion and abdominal surface displacement were limited to 4.6%±4.4% and 3.5%±5.0%, respectively. Figure 6(c) depicts the Pearson linear correlation between tumor and surrogate signals, after compensating for the measured phase shifts. Correlation coefficients averaged over all patients (median±IQR) were 0.63±0.27,0.84±0.13, and 0.82±0.15 for diaphragm, abdominal, and thoracic motion, respectively. For five out of seven patients, the highest correlation with tumor motion was obtained for the abdominal surface surrogate. Tumor–diaphragm correlation proved to be statistically lower than tumor–abdomen correlation (Wilcoxon rank sum test, p−value=0.05), but any statistical difference found was between tumor–abdomen and tumor–thorax correlation (p−value=0.3).

**Table 2:** Summary data of CBCT scans performed on lung cancer patients, including the initial angle of the treatment gantry, the duration of CBCT/AlignRT synchronization, the number of breathing cycles analyzed per patient, and the corresponding cycle length

*Patient*	*Angular Accuracy (°)*	*Real CBCT Starting Angle (°)*	*Duration of Synchronization (s)*	*Number of Breathing Cycles*	*Cycle Length (s)*
P1	0.03	−178.07	30.3	9	2.8
P2	−0.10	−178.53	31.9	5	4.6
P3	0.09	−178.50	19.2	9	1.8
P4	0.05	−178.60	34.5	8	3.3
P5	0.20	−178.27	26.3	5	3.5
P6	0.04	−178.38	29.0	6	3.3
P7	0.06	−178.61	24.6	5	3.2

## IV. DISCUSSION & CONCLUSIONS

We developed and evaluated an image‐based method to synchronize the acquisitions of X‐ray projections and optical surfaces during a CBCT scan. By avoiding the requirement of additional devices or interfaces, the reported method results in a low‐cost and easily‐implementable approach for the synchronization between in‐room X‐ray imaging and optical surface tracking. The proposed method was applied and tested on the AlignRT system, which is one of the most popular surface tracking devices in IGRT applications. However, the method is fully generalizable and can be extended to any tracking system based on optical cameras for marker‐based or markerless surface detection. The applicability of the proposed method only requires that a moving part of the CBCT/gantry unit is visible from the optical cameras for a short time window during the acquisition of the CBCT scan. The wide camera FOV, which is a specification for the use of optical tracking systems in IGRT,^(3)^ is expected to guarantee this condition both for frontal and lateral configurations of the optical cameras with respect to the couch. In the present study, the ability of the AlignRT optical system in capturing video images was exploited to track the position of the rotating CBCT/gantry component. In the case of a marker‐based tracking system, the detection of single or multiple passive markers integral with the moving component can also be exploited to implement the proposed synchronization approach.

The novel aspect of the developed method concerns the calibration procedure designed to relate the gantry angle with the position of the rotating part of the CBCT/gantry unit identified by the optical system. The proposed calibration method can be generically applied to any configuration of the CBCT and optical imaging systems, since it does not depend on the specific choice of the moving CBCT/gantry component and of the time window in which this component is visible from the optical cameras. In the present study, the calibrated transfer function model was built by acquiring the position of the CBCT flat‐panel detector at the beginning of the CBCT scan. However, the same calibration approach could be employed with any other moving part of the CBCT/gantry unit optically tracked in a different time window during CBCT acquisition. The selected tracking feature was inherent to the panel, but any external feature rigidly fixed to the CBCT/gantry unit can also be tracked. Interpolation and fitting techniques were introduced in the calibration procedure to increase the temporal resolution of the synchronization approach, overcoming the limitation due to the frame rate of the optical tracking device. The temporal accuracy assessed on a motorized moving phantom was better than 15 msec. The angular errors of the calibrated transfer function model did not exceed 0.1° in phantom tests. Comparable results were obtained also for subsequent patient CBCT acquisitions, thus confirming the long‐term validity of the calibration method implemented for CBCT/optical synchronization.

The application of the developed synchronization method to lung cancer patients allows collecting a valuable database for the analysis of the correlation and variability of breathing motion coming from multi‐modal imaging systems. Different internal and external respiratory surrogates commonly used in lung cancer radiotherapy were evaluated, including the diaphragm motion obtained from CBCT projections and the displacement of the thoracic and abdominal surface regions acquired with the optical imaging system. A large interpatient variability was found for internal and external breathing parameters. For example, the phase shifts between tumor motion and surrogate signals varied significantly among patients, as depicted in Fig. 6(b). Our results showed that the correlation between tumor motion and different breathing surrogates is also patient‐specific, indicating that it should be assessed on an individual and daily basis, as already reported elsewhere.^31^, ^37^, ^38^ In most patients the highest correlation was reached using an external surface surrogate (Fig. 6(c)). In particular, tumor motion proved to be more correlated with the displacement of the abdominal region, likely due to the higher respiratory motion amplitude compared with the thoracic region (Fig. 6(a)).^(32)^

The investigated image‐based method to synchronize the acquisition of in‐room X‐ray images and optical surfaces paves the way for a number of possible developments in the field of IGRT applications for extracranial targets. The proposed approach allows the integration of anatomical data obtained from X‐ray projections with the complementary information on the external surface motion acquired through optical systems with high spatial and temporal resolution. Real‐time surface data can be advantageously used for CBCT phase sorting, providing to each CBCT projection a breathing phase value robustly extracted from the synchronized external surface surrogate. Phase sorting is required to correct for respiratory motion in CBCT scans, allowing the reconstruction of motion‐compensated CBCT with reduced blurring artifacts and increased image quality.^(20)^ Phase sorting is also applied for the reconstruction of 4D respiratory‐correlated CBCT, which allows the verification of tumor shape and motion just prior to treatment.^19^, ^21^

The simultaneous tracking of the external surface topology and internal features provided by the proposed synchronization method can be particularly useful for tumor tracking applications. The use of breathing surrogates obtained from surface motion to infer tumor position through external‐internal correlation models has already been introduced in the clinical practice.^13^, ^14^ The developed approach might be applied to improve current tracking techniques, since it provides synchronized samples of the external surface surrogate and internal target position acquired during setup CBCT scan that could be advantageously used for the initialization of correlation models. The present study proved the potential of the developed synchronization method in estimating the variations of breathing motion parameters and external–internal correlation on an individual and daily basis, which is essential for the robustness of tumor tracking techniques. An additional application field of the proposed synchronization method might include patient‐specific breathing motion models built from CBCT and driven by an external surface surrogate,^25^, ^39^ which could be used to drive tracked radiotherapy treatments.^(28)^ The accurate knowledge of the temporal correspondence between the acquisition of X‐ray images and optical surfaces is required to ensure the effectiveness of tumor tracking methods based on external–internal correlation or surrogate‐driven motion models.
